# The Influence of Socioeconomic Status on Selection of Anticoagulation for Atrial Fibrillation

**DOI:** 10.1371/journal.pone.0149142

**Published:** 2016-02-25

**Authors:** Michelle Sholzberg, Tara Gomes, David N. Juurlink, Zhan Yao, Muhammad M. Mamdani, Andreas Laupacis

**Affiliations:** 1 Department of Medicine, St. Michael’s Hospital, Toronto, Ontario, Canada; 2 Faculty of Medicine, University of Toronto, Toronto, Ontario, Canada; 3 Department of Laboratory Medicine and Pathobiology, St. Michael’s Hospital, Toronto, Ontario, Canada; 4 Li Ka Shing Knowledge Institute of St. Michael’s Hospital, Toronto, Ontario, Canada; 5 Institute for Clinical Evaluative Sciences, Toronto, Ontario, Canada; 6 Leslie Dan Faculty of Pharmacy, University of Toronto, Toronto, Ontario, Canada; 7 Institute for Health Policy Management and Evaluation, University of Toronto, Toronto, Ontario, Canada; 8 Clinical Pharmacology and Toxicology, Sunnybrook Research Institute, Toronto, Ontario, Canada; Ottawa Hospital Research Institute, CANADA

## Abstract

**Importance:**

Without third-party insurance, access to marketed drugs is limited to those who can afford to pay. We examined this phenomenon in the context of anticoagulation for patients with nonvalvular atrial fibrillation (NVAF).

**Objective:**

To determine whether, among older Ontarians receiving anticoagulation for NVAF, patients of higher socioeconomic status (SES) were more likely to switch from warfarin to dabigatran prior to its addition to the provincial formulary.

**Design, Setting and Participants:**

Population-based retrospective cohort study of Ontarians aged 66 years and older, between 2008 and 2012.

**Exposure:**

Socioeconomic status, as approximated by median neighborhood income.

**Main Outcomes and Measure:**

We identified two groups of older adults with nonvalvular atrial fibrillation: those who appeared to switch from warfarin to dabigatran after its market approval but prior to its inclusion on the provincial formulary (“switchers”), and those with ongoing warfarin use during the same interval (“non-switchers”).

**Results:**

We studied 34,797 patients, including 3183 “switchers” and 31,614 “non-switchers”. We found that higher SES was associated with switching to dabigatran prior to its coverage on the provincial formulary (p<0.0001). In multivariable analysis, subjects in the highest quintile were 50% more likely to switch to dabigatran than those in the lowest income quintile (11.3% vs. 7.3%; adjusted odds ratio 1.50; 95% CI 1.32 to 1.68). Following dabigatran’s addition to the formulary, the income gradient disappeared.

**Conclusions and Relevance:**

We documented socioeconomic inequality in access to dabigatran among patients receiving warfarin for NVAF. This disparity was eliminated following the drug’s addition to the provincial formulary, highlighting the importance of timely reimbursement decisions.

## Background

Third party insurers generally decide which drugs they will reimburse based upon an assessment of value for money. Many drugs are fully funded, others are reimbursed only for patients who fulfill eligibility criteria, and others are not funded at all.[1, 2] Patients often pay out-of-pocket for drugs not covered by a third party.

Supporters of this approach to drug reimbursement note that all patients have equal access to drugs felt to represent good value for money, and that public resources are not well spent on drugs not deemed cost-effective.[2–5] However, critics suggest that it can take time for public plans to incorporate new evidence about a drug’s benefits in their decision-making. They argue that, in these instances, less affluent patients are less able to access effective new drugs when the cost incurs economic hardship.[6, 7] Data from publicly funded drug programs may provide insight into this issue.

For decades, many patients with atrial fibrillation have been anticoagulated with vitamin K antagonists (VKAs) such as warfarin, to diminish their risk of arterial thromboembolism, particularly stroke. The introduction of the direct oral anticoagulants (DOACs) as alternatives to VKAs has been met with cautious enthusiasm among clinicians [8]. The major advantages of the DOACs include their rapid onset of action, shorter half-lives, lack of requirement for regular laboratory monitoring and the absence of food interactions when compared to VKAs.[9, 10] DOACs are now recommended for the prevention and treatment of thromboembolism. Currently available DOACs include dabigatran which directly inhibits the final effector of coagulation, thrombin (factor IIa), while rivaroxaban and apixaban directly inhibit the rate-limiting enzyme of coagulation, factor Xa. The RE-LY multicenter, non-inferiority trial, which compared the use of dabigatran with warfarin for nonvalvular atrial fibrillation (NVAF), found essentially similar rates of stroke or systemic embolism but lower rates of life-threatening and major bleeding with dabigatran [9, 10].

In Canada, dabigatran etexilate was approved by Health Canada on October 26^th^, 2010, for thromboprophylaxis in patients with NVAF. It was added to the provincial formulary almost 18 months later, on April 24^th^, 2012. We sought to determine whether older Ontarians who switched from warfarin to dabigatran during this period were more likely to live in wealthier neighborhoods, as compared with those who remained on warfarin. A secondary objective tested whether any identified socioeconomic gradient persisted once dabigatran became available through the public drug program.

## Methods

We conducted a population-based cohort study of Ontarians aged 66 and older with NVAF who were treated with warfarin between October 28, 2008, and October 26, 2010. Patient information was anonymized and de-identified prior to analysis. Written informed consent was not given by participants for their records to be used in this study. This study was approved by the Research Ethics Board of Sunnybrook Health Sciences Centre, Toronto, Ontario.

### Data Sources

We used the Ontario Registered Persons Database (RPDB), which contains basic demographic data and information on vital status, to identify SES and urban or rural patient residence.[11–13] The Ontario Drug Benefit Program (ODBP) database was used to identify prescriptions for medications. We used the Canadian Institute for Health Information Discharge Abstract Database (CIHI-DAD), the National Ambulatory Care Reporting System (NACRS) and the Ontario Health Insurance Plan (OHIP) database to identify patients with NVAF and other comorbidities (including major hemorrhage). These datasets were linked using unique encoded identifiers and analyzed at the Institute for Clinical Evaluative Sciences (ICES).

We approximated SES based on each patient’s place of residence on October 26, 2010, (the date of Health Canada approval for dabigatran), and patients were divided into quintiles according to median neighborhood income, as done before.[14–16] Previous studies have suggested strong, consistent, and progressive income-related differences in drug treatment selection and we hypothesized that the same is true for the selection of anticoagulant therapy for NVAF in Ontario, Canada.[17–20] The association between the SES and dabigatran prescription seems plausible because the cost of dabigatran in Ontario is approximately 20-fold higher than warfarin, and this association has previously been described in the United States.[20, 21]

#### Study Subjects

**Fig 1:**
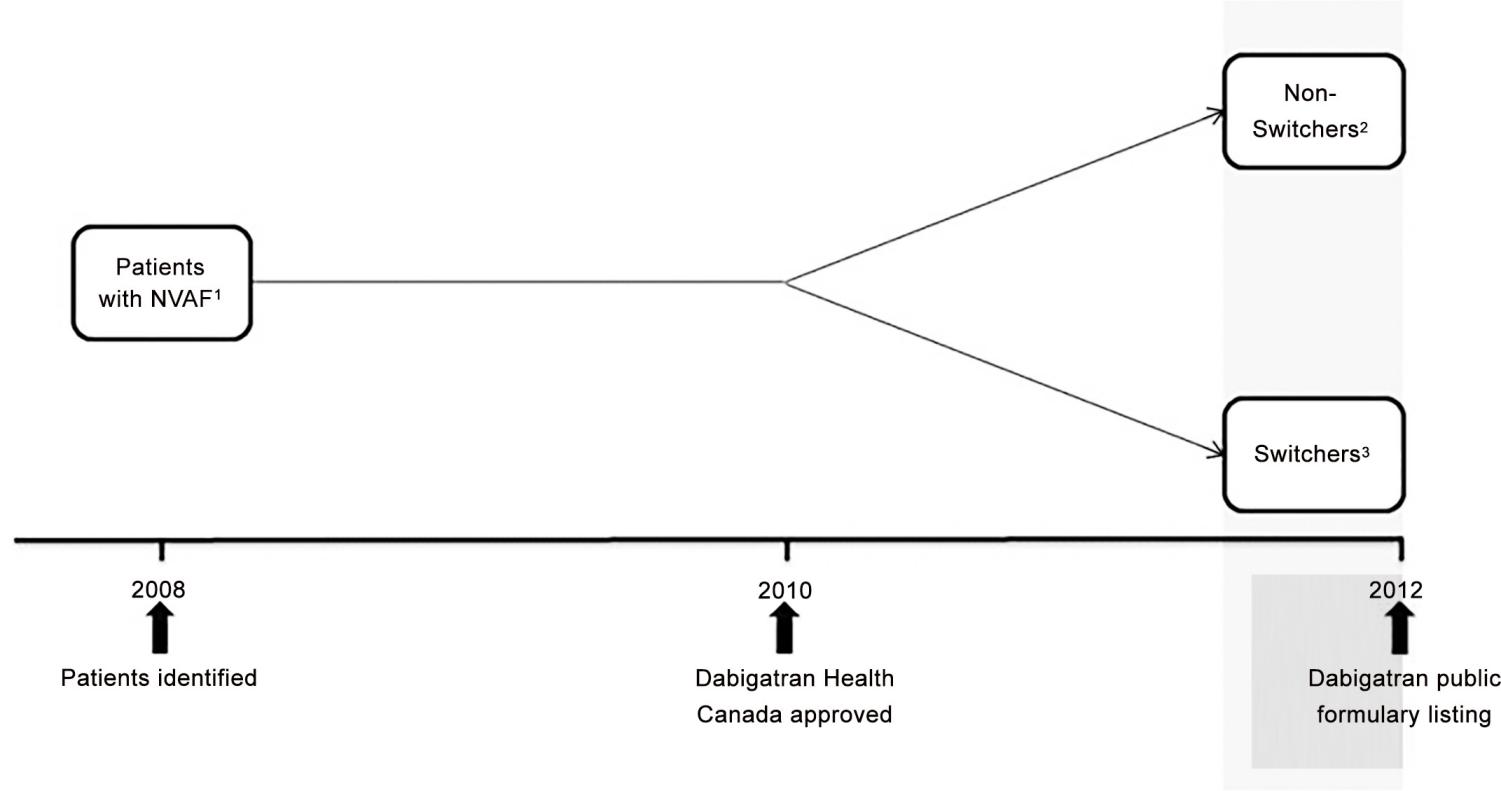
Study Design. Legend: ^1^ Patient with Non-Valvular Atrial Fibrillation on warfarin over the age of 66 years.^2^ Non-switchers are those with ≥ 2 warfarin prescriptions in the 6 months prior to dabigatran being listed on the public formulary. ^3^ Switchers are those with NO warfarin prescriptions in the 6 months prior to dabigatran being listed on the public formulary.

We developed a cohort of Ontarians with NVAF aged 66 years and older treated with warfarin in the two years prior to Health Canada’s approval of dabigatran, which occurred on October 26, 2010 (Fig 1). Patients were identified on the basis of an inpatient hospitalization or emergency department visit for atrial fibrillation using the International Classification of Diseases and Related Health Problems, 10th revision codes (ICD-10) I48.0 and I48.1, or a physician visit with the diagnosis of atrial fibrillation (OHIP diagnosis code 427) in the context of previous warfarin use. Validation studies suggest ICD codes for the diagnosis of atrial fibrillation have a positive predictive value between 70 and 96%.[22]

To focus on patients with evidence of ongoing warfarin use, we limited our analysis to patients with two or more warfarin prescriptions in the 6 months before Health Canada’s approval of dabigatran. We did not include patients with only one warfarin prescription, those with mitral stenosis, prosthetic heart valves, or mitral or aortic valve surgery, as well as residents of chronic care facilities in the three years prior to formulary listing of dabigatran.[23] We also excluded patients who died prior to the end of the study period (i.e. ineligible for early or late switch) and those with a hemorrhage leading to a hospital visit in the three years prior to cohort entry or during follow-up, to avoid the possibility that anticoagulant choice was influenced by a previous hemorrhage. The ICD-10 codes used for the definition of major hemorrhage are detailed in S1 Appendix, and have a positive predictive value of 87% and a negative predictive value of 92% for the identification of major bleeding events.[24] Patients with active liver disease, severe stroke and a creatinine clearance <30ml/min were excluded from the RE-LY study; we could not exclude them because these variables were not part of our databases.

#### Outcome Definition

We stratified patients into two mutually exclusive groups. We defined “switchers” as patients whose warfarin use ceased in the interval between dabigatran’s approval by Health Canada and its inclusion on the provincial formulary, and who had evidence of dabigatran use within two years thereafter. We defined “non-switchers” as patients who continued warfarin therapy during the same interval, and had ongoing use of warfarin even after dabigatran’s inclusion on the public formulary.

We did not have data about drugs paid for privately, either out-of-pocket or by private insurance companies. Because “switchers” were patients receiving warfarin for NVAF who later transitioned to dabigatran, we inferred that discontinuation of warfarin in the interval between dabigatran’s approval and formulary listing reflected institution of dabigatran during this same period.

In a secondary analysis, we stratified “non-switchers” into those who initially remained on warfarin, but transitioned to dabigatran in the six months following its addition to the ODBP formulary (“late switchers”) and those who continued to use warfarin exclusively (“continuous warfarin users”; Fig 1), to explore whether addition of dabigatran to the provincial formulary attenuated any SES gradient identified in our primary analysis. The initial starting dose of dabigatran (110 mg versus 150 mg) was examined for “switchers” and “late switchers”.

#### Statistical Analysis

We used the Cochran-Armitage trend test to assess for a linear trend in SES quintile among “switchers” and “non-switchers”, and used logistic regression to control for relevant covariates and to examine the independent effect of income quintile on early adoption of dabigatran. We adjusted for demographic variables, comorbid illnesses, medication-related variables and any specialist visits between Health Canada approval and inclusion of dabigatran on the provincial formulary (S2 Appendix). Covariates were included in the model if we deemed them clinically important, if the standardized mean difference (reflecting the mean difference as a percentage of the standard deviation) exceeded 0.1 between any income quintiles comparison, or in the event of subjective evidence of trending incremental or decremental variation across income quintiles [25] Covariates included in the multivariable adjustment were age, gender, non-rural residence, Charlson Comorbidity Index, myocardial infarction, cerebrovascular disease, diabetes mellitus, renal disease, specialist visit, aspirin use, clopidogrel use and number of drugs received in the previous year (Table 1; Table 2).

**Table 1:** Primary Analysis: Odds of Switching to Dabigatran Prior to ODBP listing of Dabigatran–Adjusted Model.

Odds Ratio Estimates	
Effect	Point Estimate (95% Confidence Limits)
*Income Quintile Comparisons*	
Income Quintile 2 vs 1	1.13 (0.99–1.28)
Income Quintile 3 vs 1	1.19 (1.05–1.36)
Income Quintile 4 vs 1	1.28 (1.13–1.45)
Income Quintile 5 vs 1	1.50 (1.32–1.68)
*Demographics*	
Age	0.97 (0.96–0.97)
Female	0.92 (0.86–1.00)
Non Rural residence	1.26 (1.13–1.42)
*Comorbidity–Past 3 years*	
Myocardial infarction	1.04 (0.83–1.30)
Cerebrovascular Disease	1.04 (0.89–1.22)
Diabetes mellitus	0.87 (0.80–0.95)
Renal disease	0.63 (0.55–0.73)
*Charlson Comorbidity Index*	
Charlson Score 0	1.14 (1.04–1.26)
Charlson Score 1	1.01 (0.89–1.15)
Charlson Score ≥2	0.75 (0.66–0.86)
*Specialist Visit*	
Cardiologist Visit	1.74 (1.59–1.90)
Neurologist Visit	1.36 (1.21–1.53)
*Antiplatelet Drug Use in Past 120 Days*	
Aspirin use	1.15 (0.80–1.65)
Clopidogrel use	1.24 (1.00–1.55)
NSAID use	1.06 (0.92–1.22)
Number of drugs in past 1 year	0.98 (0.98–0.99)

**Table 2:** Secondary Analysis: Odds of Switching to Dabigatran after ODBP listing of Dabigatran–Adjusted Model.

Odds Ratio Estimates	
Effect	Point Estimate (95% Confidence Limits)
*Income Quintile Comparisons*	
Income Quintile 2 vs 1	0.92 (0.82–1.04)
Income Quintile 3 vs 1	0.92 (0.81–1.04)
Income Quintile 4 vs 1	0.92 (0.81–1.04)
Income Quintile 5 vs 1	0.93 (0.82–1.05)
*Demographics*	
Age	0.99 (0.98–0.99)
Female	0.99 (0.91–1.07)
Non Rural residence	1.23 (1.10–1.39)
*Comorbidity–Past 3 years*	
Myocardial infarction	1.09 (0.87–1.36)
Cerebrovascular Disease	1.16 (1.00–1.36)
Diabetes mellitus	0.89 (0.81–0.97)
Renal disease	0.71 (0.62–0.81)
*Charlson Comorbidity Index*	
Charlson Score 0	1.02 (0.92–1.14)
Charlson Score 1	0.92 (0.81–1.05)
Charlson Score ≥2	0.88 (0.78–1.00)
*Specialist Visit*	
Cardiologist Visit	1.07 (0.98–1.16)
Neurologist Visit	1.04 (0.91–1.19)
*Antiplatelet Drug Use in Past 120 Days*	
Aspirin use	1.24 (0.87–1.76)
Clopidogrel use	0.68 (0.52–0.90)
NSAID use	1.16 (1.01–1.34)
Number of drugs in past 1 year	1.03 (1.02–1.03)

To assess our secondary objective, we used the same statistical methods to investigate the relationship between increasing income quintile and selection of anticoagulant in the “late switcher” versus “continuous warfarin users” strata during the six months after dabigatran ODBP formulary listing.

Results from the Cochran-Armitage trend test were considered statistically significant if the p value was <0.05. The Bonferroni method was used to account for multiple hypothesis testing.[26] All analyses were performed using SAS statistical software, version 9.2 (SAS Institute Inc, Cary, North Carolina).

## Results

We identified 34,797 older Ontarians with NVAF treated with warfarin, including 31,614 “non-switchers” and 3,183 “switchers”. The characteristics of patients are described in Table 3.

**Table 3:** Baseline Characteristics of Elderly Ontarians with Non-Valvular Atrial Fibrillation.

		Income Quintiles Based on Median Neighborhood Income (Lowest to Highest)				
Characteristic	All Participants	1	2	3	4	5
No. of participants	34797	6340	7245	6827	7023	7362
*Demographics*						
Age, years^a^	78.6	78.7 (6.8)	78.7 (6.6)	78.4 (6.7)	78.4 (6.8)	78.8 (6.9)
Female (%)	17156 (49.3)	3545 (54.5)	3716 (51.3)	3226 (47.3)	3270 (46.6)	3399 (46.2)
Rural residence (%)	5081 (14.6)	1074 (16.9)	1071 (14.8)	1037 (15.2)	968 (13.8)	931 (12.6)
*Comorbidity–past 3 years*						
MI (%)	1251 (3.6)	281 (4.4)	246 (3.4)	242 (3.5)	274 (3.5)	208 (2.8)
CVD (%)	2480 (7.1)	488 (7.7)	522 (7.2)	504 (7.4)	497 (7.1)	469 (6.4)
PVD (%)	765 (2.2)	155 (2.4)	149 (2.1)	168 (2.5)	148 (2.1)	145 (2.0)
Hepatic disease (%)	503 (1.4)	82 (1.3)	111 (1.5)	98 (1.4)	108 (1.5)	104 (1.4)
Renal disease (%)	3969 (11.4)	796 (12.6)	865 (11.9)	787 (11.5)	786 (11.2)	735 (10.0)
Diabetes mellitus (%)	11983 (34.4)	2454 (38.7)	2653 (36.6)	2410 (35.3)	2303 (32.8)	2163 (29.4)
*Charlson Comorbidity Index*^b^						
Charlson 0 (%)	5886 (16.9)	1006 (15.9)	1224 (16.9)	1155 (16.9)	1193 (17.0)	1308 (17.8)
Charlson 1 (%)	4236 (12.2)	850 (13.4)	861 (11.9)	869 (12.7)	823 (11.7)	833 (11.3)
Charlson ≥ 2 (%)	5776 (16.6)	1190 (18.8)	1279 (17.7)	1156 (16.9)	1136 (16.2)	1015 (13.8)
No admission to hospital (%)	18899 (54.3)	3294 (52.0)	3881 (53.6)	3647 (53.4)	3871 (55.1)	4206 (57.1)
*Specialist Visit*^c^						
Cardiologist (%)	23593 (67.8)	4146 (65.4)	4857 (67.0)	4546 (66.6)	4859 (69.2)	5185 (70.4)
Neurologist (%)	3079 (8.8)	538 (8.5)	631 (8.7)	561 (8.2)	626 (8.9)	723 (9.8)
*Medication use in past 120 days*						
*Antiplatelet Drugs*						
ASA (%)	344 (1.0)	83(1.3)	82 (1.1)	66 (1.0)	65 (0.9)	48 (0.7)
Clopidogrel (%)	954 (2.7)	163 (2.6)	205 (2.8)	200 (2.9)	207 (2.9)	179 (2.4)
Dipyridamole/ASA (%)	79 (0.2)	12 (0.2)	16 (0.2)	21 (0.3)	16 (0.2)	14 (0.2)
Prasugrel (%)	0 (0)	0 (0)	0 (0)	0 (0)	0 (0)	0 (0)
Ticlopidine (%)	0 (0)	0 (0)	0 (0)	0 (0)	0 (0)	0 (0)
NSAID (%)	2525 (7.3)	465 (7.3)	530 (7.3)	521 (7.6)	495 (7.0)	514 (7.0)
*Drugs thought to interact with dabigatran*						
Quinidine (%)	0 (0)	0 (0)	0 (0)	0 (0)	0 (0)	0 (0)
Amiodarone (%)	2199 (6.3)	406 (6.4)	467 (6.4)	404 (5.9)	454 (6.5)	468 (6.4)
Ketoconazole (%)	210 (0.6)	43 (0.7)	47 (0.6)	44 (0.6)	40 (0.6)	36 (0.5)
Verapamil (%)	507 (1.5)	98 (1.5)	113 (1.6)	99 (1.5)	101 (1.4)	96 (1.3)
Rifampicin (%)	0 (0)	0 (0)	0 (0)	0 (0)	0 (0)	0 (0)
Number of Drugs in past 1 year^a^	10.8	11.5 (5.5)	11.0 (5.3)	10.9 (5.2)	10.5 (5.0)	10.1 (4.9)

Abbreviations: IQ, income quintile; MI, myocardial infarction; CVD, cerebrovascular disease; PVD, peripheral vascular disease, ASA, acetylsalicylic acid, NSAID, non-steroidal anti-inflammatory drug.

^a^Values are means (SD).

^b^Charlson Comorbidity Index–comorbidity measure

^c^ Specialist visit–any cardiologist (or neurologist) visit in the time between Health Canada approval and dabigatran listing on the Ontario Drug Benefit formulary

In the primary analysis, we found a significant association between increasing SES and switching to dabigatran in the interval between its market approval and inclusion on the provincial formulary (p<0.0001; Table 4). In the highest income quintile, 11.4% of patients switched to dabigatran during that time, compared to only 7.3% in the lowest income quintile (adjusted odds ratio 1.50; 95% confidence interval 1.33 to 1.69; Table 1). Other predictors of early switching to dabigatran included younger age, absence of renal disease or diabetes, lower Charlson comorbidity index, fewer concomitant medications, clopidogrel use, provision of care by a cardiologist or neurologist, and urban place of residence (Table 1; S4 Appendix). In the model, our conclusions did not change after application of the Bonferroni correction.

**Table 4:** Assessment for Trend of Switchers across Income Quintiles Prior to ODBP listing of Dabigatran.

Income Quintile	Non-Switchers	Switchers
1—lowest	92.7%	7.3%
2	91.7%	8.4%
3	91.1%	8.9%
4	90.3%	9.7%
5—highest	88.7%	11.3%

Cochran-Armitage Trend Test, one sided p value <0.0001.

In the secondary analysis, the 31,614 “non-switchers” were categorized into 28,745 patients who remained on warfarin after dabigatran was listed by the ODBP and 2,869 patients who transitioned to dabigatran in the subsequent 6 months. We found no statistically significant association between income quintile and switching to dabigatran after the drug’s addition to the provincial formulary (8.9% switched in the highest vs. 9.7% in the lowest income quintile, p = 0.085 and adjusted OR 0.93 {95% CI 0.82 to 1.05) among individuals who had remained on warfarin prior to dabigatran’s addition to the provincial formulary (Table 2; S3 and S5 Appendix).

The majority of “switchers” (N = 1942, 61.0%) and “late switchers” (N = 2056, 71.7%) received the lower 110 mg dose of dabigatran rather than the 150 mg dose.

## Discussion

In this population-based study spanning four years, we found that individuals of higher SES receiving warfarin for NVAF were more likely to switch to dabigatran in the period between its market approval and inclusion on the provincial formulary. Importantly, this gradient was eliminated after the drug became available through public reimbursement. These findings accord with a recent retrospective claims analysis that found that those with an enriched health plan were more likely to use dabigatran, and with other studies that suggest SES can influence medication use.[27–32]

While the finding that SES might influence access to uninsured medications is not surprising, our study characterizes the magnitude of this effect in a publicly funded system. Patients living in the highest income quintile neighborhoods were 50 percent more likely to use dabigatran than those living in the lowest income quintile neighborhoods.

Access to specialist care is affected by patient sociodemographic and economic status.[33, 34] In our study, the observation that patients under the care of cardiologists or neurologists were more likely to receive dabigatran is not surprising because of the marketing of direct oral anticoagulants (DOACs) by the pharmaceutical industry to specialist physicians, as well as the likelihood that specialists were more familiar with early data supporting the use of dabigatran in NVAF, compared with general practitioners.[27] Previous studies have found that specialists are more likely than general practitioners to prescribe new drugs. [35, 36]

Although debate exists with regard to its place relative to other DOACs, dabigatran has been shown to be a cost-effective thromboprophylactic strategy relative to warfarin.[37–42] The low number of “switchers” to dabigatran, whether early or late, was not unexpected. We believe that this is reflective of uncertainty regarding the use of dabigatran for NVAF and is in keeping with the cautious early uptake of dabigatran identified in the American Outcomes Registry for Better Informed Treatment of Atrial Fibrillation (ORBIT-AF) study [21]. The higher proportion of individuals in both groups who received lower dose dabigatran likely indicates physician vigilance with dabigatran use in our older study patient population[21].Nonetheless, our findings support the need to ensure up-to-date cost-effectiveness reviews of new drugs, and a timely mechanism to incorporate the results of those reviews into formulary decision making, to prevent disadvantaged access among less affluent patients.

Strengths of our study include its large population-based sample, and the high accuracy of administrative databases for determining drug use and the diagnosis of atrial fibrillation coupled with concomitant anticoagulant use.We obtained concordant results from two different analyses, the Cochran-Armitage trend test and logistic regression, enhancing the robustness of our findings.

This study has several limitations. First, we lacked access to claims data for dabigatran prior to its inclusion on the provincial formulary. We address this limitation by using a strict definition of continuous warfarin use and documentation of dabigatran use in “switchers” after its public formulary listing. Second, neighborhood income was used as a surrogate for patient SES because the administrative databases do not have measures of income at the level of the individual patient. However, this approach has previously been validated as a measure of household income and has been used in many other population-based studies.[12, 13, 43] Third, we could not exclude some patients who were excluded from the RE-LY study due to the constraints of the provincial databases. Therefore, selection bias could have influenced the association found between anticoagulant selection and the absence of renal disease since those who may have had an absolute contraindication to dabigatran (i.e. creatinine clearance < 30 ml/min) were included in the study. However, this does not affect the main analysis assessing the association between SES and prescription of dabigatran. Fourth, we restricted this study to dabigatran because the other DOACs were either not yet approved for use in Canada or not listed on the Ontario formulary at the time of analysis. Finally, we did not have prescription data on younger Ontarians; however, NVAF is principally a disorder of older patients.

Our findings likely apply to many other medications that represent good value for money, so delays in third-party medication coverage collectively have much larger impacts than we have demonstrated for dabigatran alone.[44] While we believe a thorough assessment of a drug’s cost-effectiveness is critical to the sustainability of a healthcare system, we also recognize a need to make this process as efficient as possible to avoid unnecessary delays in patient access to truly effective and cost-effective therapies. Our findings should be a stimulus for Canadian publicly funded drug plans to review their processes to ensure high levels of efficiency, especially removing any duplication in reviews of cost-effectiveness conducted by the national and provincial review panels, expediting price negotiation and ensuring that decisions are made as soon as all the necessary information is available.

In conclusion, we documented socioeconomic inequality in access to dabigatran among NVAF patients receiving warfarin. This finding supports the need to update public formularies in a timely manner based upon high quality evidence about cost-effectiveness. We recognize that a drug can be cost-effective while at the same time associated with considerable costs to payers, either because the drug itself is expensive or because of its use by many patients. However, equity is an important principle in health care and should influence reimbursement policy so that the poor are not required to pay more for prescription drugs as a proportion of their income compared to the rich.[1, 44–47]

## Supporting Information

S1 Appendix Diagnosis codes used to define hemorrhage outcomes. (DOCX)

S2 Appendix Covariates adjusted for within Logistic Regression Model. (DOCX)

S3 Appendix Assessment for Trend of Switchers across Income Quintiles After ODBP listing of Dabigatran. (DOCX)

S4 Appendix Forest Plot of Primary Analysis: Odds of Switching to Dabigatran Prior to ODBP listing of Dabigatran–Adjusted Model. (DOCX)

S5 Appendix Forest Plot of Secondary Analysis: Odds of Switching to Dabigatran after ODBP listing of Dabigatran–Adjusted Model. (DOCX)
