# Supplementary material for: The Influence of Socioeconomic Status on Selection of Anticoagulation for Atrial Fibrillation
Source: PLoS One. 2016 Feb 25;11(2):e0149142. doi: 10.1371/journal.pone.0149142 (PMC4767939; doi:10.1371/journal.pone.0149142)
Supplement: S1 Appendix — (DOCX) [file pone.0149142.s001.docx]

**APPENDIX:**

**S1 Appendix. Diagnosis codes used to define hemorrhage outcomes**

| **Hemorrhage Type** | **ICD-10 Codes** |
| --- | --- |
| Intracerebral | I60, I61, I62.0, I62.1, I62.9 |
| Upper Gastrointestinal | K92.0, K92.1, I85.0, I98.20, I98.3, K22.10, K22.12, K22.14, K22.16, K25.0, K25.2, K25.4, K25.6, K26.0, K26.2, K26.4, K26.6, K27.0, K27.2, K27.4, K27.6, K28.0, K28.2, K28.4, K28.6, K29.0, K63.80, K31.80 |
| Lower Gastrointestinal | K55.20, K62.5, K92.2 |
| Other Hemorrhage | N02.0, N02.1, N02.2, N02.3, N02.4, N02.5, N02.6, N02.7, N02.8, N02.9, K66.1, N93.8, N93.9, N95.0, R04.1, R04.2, R04.8, R04.9, R31.0, R31.1, R31.8, R58, D68.3, H35.6, H43.1, H45.0, M25.0 |
