# Supplementary material for: The Influence of Socioeconomic Status on Selection of Anticoagulation for Atrial Fibrillation
Source: PLoS One. 2016 Feb 25;11(2):e0149142. doi: 10.1371/journal.pone.0149142 (PMC4767939; doi:10.1371/journal.pone.0149142)
Supplement: S2 Appendix — (DOCX) [file pone.0149142.s002.docx]

**S2 Appendix. Covariates adjusted for within Logistic Regression Model**

| **Covariate Category** | **Specific Covariates** |
| --- | --- |
| Demographics | - Age - Gender - Urban vs. rural residence |
| Comorbid Illnesses | - Renal disease - Hepatic disease - Myocardial infarction - Cerebrovascular disease - Peripheral vascular disease - Diabetes mellitus - Charlson Comorbidity Index |
| Medication Related | - Number of drugs prescribed in the last year prior to cohort entry - Antiplatelet agents [acetylsalicylic acid ([37](#_ENREF_37)), clopidogrel, dipyridamole/ASA, ticlopidine, prasugrel or nonsteroidal anti-inflammatory drugs [NSAIDs] - Prescription of drugs with the potential to influence dabigatran levels [quinidine, amiodarone, ketoconazole, verapamil, rifampin] |
| Specialist Visit | - Neurologist visit - Cardiologist visit |
