# Supplementary material for: The Influence of Socioeconomic Status on Selection of Anticoagulation for Atrial Fibrillation
Source: PLoS One. 2016 Feb 25;11(2):e0149142. doi: 10.1371/journal.pone.0149142 (PMC4767939; doi:10.1371/journal.pone.0149142)
Supplement: S3 Appendix — (DOCX) [file pone.0149142.s003.docx]

**S3 Appendix. Assessment for Trend of Switchers across Income Quintiles After ODBP listing of Dabigatran**

| **Income Quintile** | **Non-Switchers** | **Switchers** |
| --- | --- | --- |
| 1 - lowest | 90.3% | 9.7% |
| 2 | 91.0% | 8.9% |
| 3 | 91.1% | 8.9% |
| 4 | 91.1% | 8.8% |
| 5 - highest | 91.1% | 8.9% |

**Cochran-Armitage Trend Test, one sided p value=0.085**
