# Supplementary material for: The Influence of Socioeconomic Status on Selection of Anticoagulation for Atrial Fibrillation
Source: PLoS One. 2016 Feb 25;11(2):e0149142. doi: 10.1371/journal.pone.0149142 (PMC4767939; doi:10.1371/journal.pone.0149142)
Supplement: S4 Appendix — (DOCX) [file pone.0149142.s004.docx]

**S4 Appendix. Forest Plot of Primary Analysis: Odds of Switching to Dabigatran Prior to ODBP listing of Dabigatran – Adjusted Model**


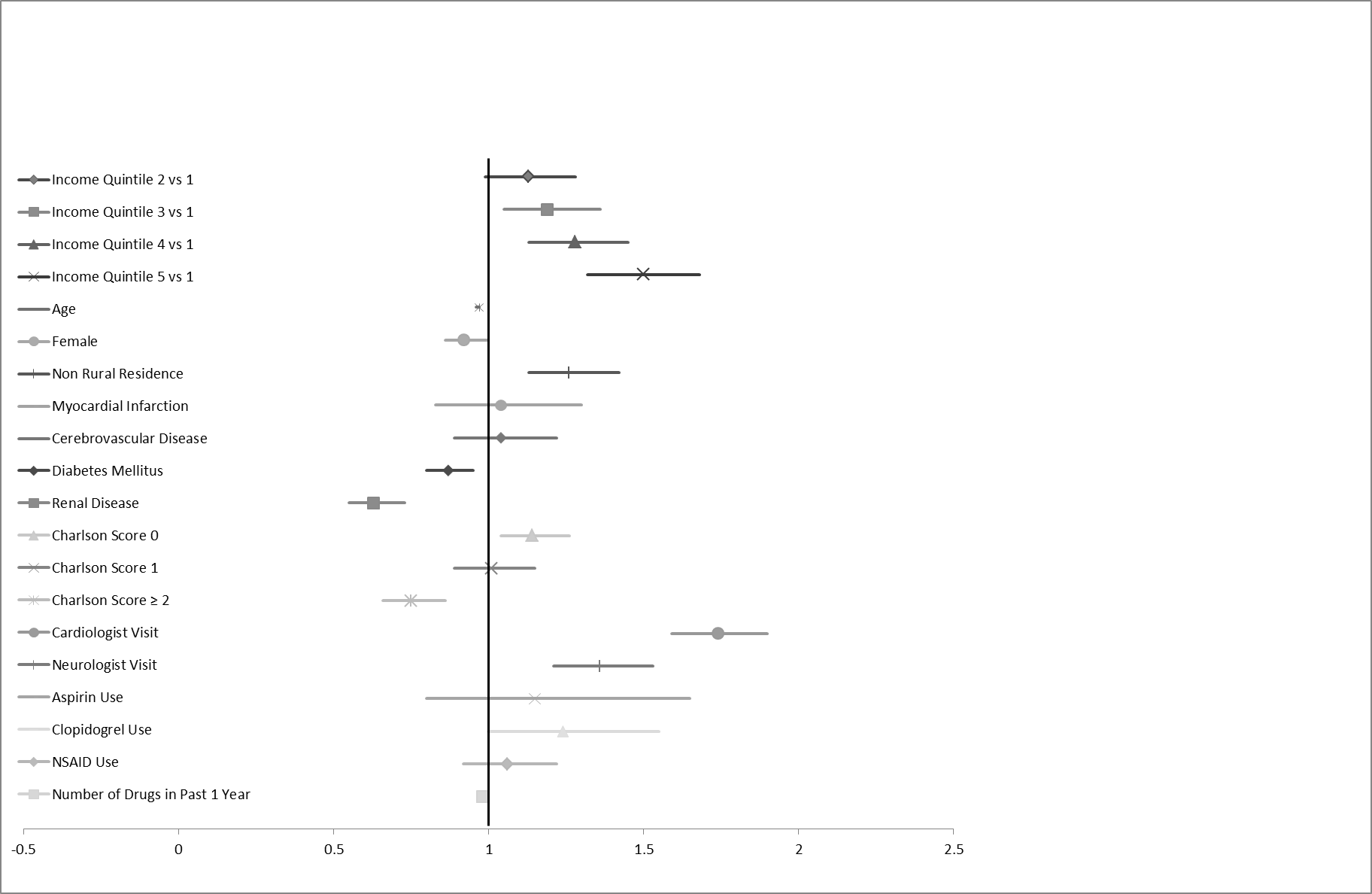


Point estimate (odds ratio) in center for each variable with surrounding 95% confidence interval.
